# Therapeutic Implications of microRNAs in Depressive Disorders: A Review

**DOI:** 10.3390/ijms232113530

**Published:** 2022-11-04

**Authors:** Mubashir Hassan, Aqsa Amir, Saba Shahzadi, Andrzej Kloczkowski

**Affiliations:** 1 The Steve and Cindy Rasmussen Institute for Genomic Medicine, Nationwide Children Hospital, Columbus, OH 43205, USA; 2 Institute of Molecular Biology and Biotechnology, The University of Lahore, Defense Road Campus, Lahore 54590, Pakistan; 3 Department of Pediatrics, The Ohio State University, Columbus, OH 43205, USA

**Keywords:** depression, MicroRNA, depressive disorder, anxiety

## Abstract

MicroRNAs are hidden players in complex psychophysical phenomena such as depression and anxiety related disorders though the activation and deactivation of multiple proteins in signaling cascades. Depression is classified as a mood disorder and described as feelings of sadness, loss, or anger that interfere with a person’s everyday activities. In this review, we have focused on exploration of the significant role of miRNAs in depression by affecting associated target proteins (cellular and synaptic) and their signaling pathways which can be controlled by the attachment of miRNAs at transcriptional and translational levels. Moreover, miRNAs have potential role as biomarkers and may help to cure depression through involvement and interactions with multiple pharmacological and physiological therapies. Taken together, miRNAs might be considered as promising novel therapy targets themselves and may interfere with currently available antidepressant treatments.

## 1. Introduction 

Anxiety is a disturbed mental health condition in which a person responds to stimuli and situations with fear, increased heart rate, and sweating [1]. There are different types of anxiety disorders at diverse age ranges (16–85 years) such as generalized anxiety (GA; 2.7%), obsessive-compulsive anxiety (OC; 1.9%), panic (2.6%), social anxiety (4.7%), post-traumatic stress disorder (PTSD; 6.4%) [2]. Globally, it has been estimated that 264 million individuals are facing anxiety problems which are mostly affecting females as compared to males (4.6%, vs. 2.6%). The major cause of anxiety disorder is disturbance in brain chemistry, environmental stress, and genetic inheritance from parents [3]. Similarly, as anxiety, depression is also a neurological disease in which person feels hopeless, sleeps too much, thinks about death and suicide, and feels guilty [4]. There are different types of depression such as major depressive disorder (MDD), persistent depressive disorder (PDD)—a depression stage which lasts for two or more years, and disruptive mood dysregulation disorder (DMDD) that frequently becomes aggressive [5]. The major causes of depression are: genetic predispositions, poor nutrition, stress, excessive intake of drugs, and brain unbalancing. However, it has also been observed that around 85% of depression patients experience significant symptoms of anxiety [6]. 

## 2. Neurobiology and Molecular Basis of Anxiety and Depression

The etiology and underpinning of anxiety and depression depends upon the neuronal correlations in brain and their responses to different biological conditions and environmental factors [7]. The disturbed behavior such as fear and stress depends upon the neuronal activity and communication between the related brain areas such as hippocampus (the memory center), anterior cingulate (the brain’s conflict-resolution area), and prefrontal cortex (involved in planning and executing activities), respectively [8,9]. The basic features of anxiety are identification of potential threat or harm, the psychological features of alarm, dread or fear, and physiological response that includes autonomic discharge and motor activity, respectively. However, each feature is controlled by different brain regions such as cortical, subcortical and hypothalamus, respectively. It has been also observed that hypothalamus is particularly involved in alerting the person of impending harm, and brain stem responses [10]. Moreover, the individuals with panic disorder may have subtle developmental abnormalities of the right cerebral hemisphere. The left cerebral hemisphere specializes in discrete functions like language, while the right hemisphere specializes in multimodal sensory function and emotions. Lesions in right hemisphere, i.e., mesial temporal cortex are commonly associated with panic disorder [10]. Amygdala consists of two sub-regions such as basolateral amygdala (BLA) and central amygdala (CeA) which is usually linked with emotional response to environmental information [9]. The medial prefrontal cortex (MPFC) is connected to the hippocampus and encodes anxiety related behavior [9]. 

## 3. Disruptions of Synaptic Circuits and Their Associated Function

Disturbances in synaptic circuits are caustic factors of depression and related disorders [11,12]. Reduction in prefrontal cortex and hippocampus volume associates with ailment and time of action and chronic conditions in depression. When stress is introduced, it results in the reduction or loss of neurons and glia in the hippocampus and prefrontal cortex (PFC) [13,14,15]. The synaptic plasticity of the brain (the change that occurs at synapses, the junctions between neurons that allow them to communicate) plays significant function in signaling pathways and synaptogenesis. The synaptic plasticity is also involved in decrease or loss of neurotrophic factors (NTFs), disturbance of estradiol cycle, and increased levels of inflammatory cytokines which are ultimately causes of depression [13]. Glia (non-neuronal cells) also plays an important role in the regulation and reuptake of glutamate which is also involved in the depression [16,17]. 

## 4. Hypothalamic-Pituitary-Adrenal (HPA) Axis and Glucocorticoid

In depression, acute traumatic or chronic stress is considered the most common factor [18], however, long-lasting stress can cause changes in DNA and histones which results in abnormal behavior in adulthood [19,20,21]. Stress responses can be activated by the hypothalamic-pituitary-adrenal (HPA) axis due to increase in the circulating levels of glucocorticoids. High levels of glucocorticoid affect multiple organs and lead to negative feedback mechanism [22,23,24]. 

**Figure 1:**
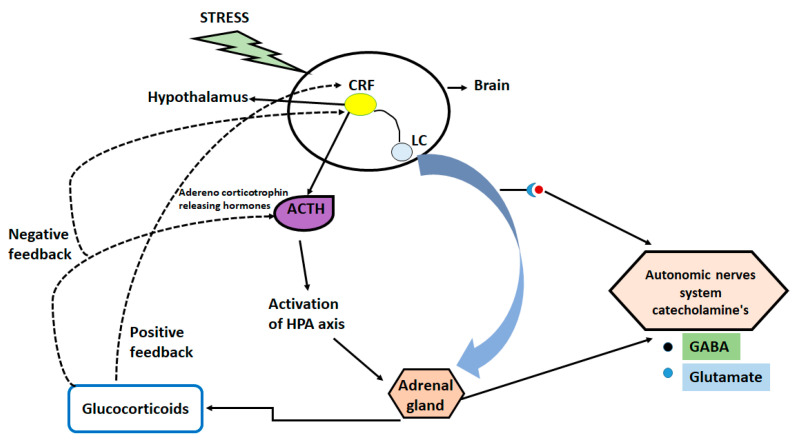
Mechanistic pathway of stress induced disturbance in brain.

The increased glucocorticoid levels act on multiple levels of neuronal functions (decreasing levels or numbers of synapses) and behaviors [25,26]. The prior research also showed that acute stress increases the levels of glutamate and neuronal transmission in the prefrontal cortex and hippocampus, thereby influencing some aspects of cognitive processing [17,27,28]. Moreover, the molecular studies from mouse and human reveal the role of glucocorticoid in the regulation of signaling pathways, gene transcription, and epigenetic mechanism of regulation of glucocorticoid receptors [29]. Additionally, genetic variations in the genes encoding the FKBP5 and CRF-1, have been linked with different neuropsychiatric diseases and stressful events of life such as childhood abuse and trauma [30]. Figure 1 illustrates the mechanistic pathway of stress induced disturbance in brain.

## 5. Neurotropic Factors and Associated Signaling Pathways in Stress and Depression

There are several growth factors, including brain-derived neurotrophic factors (BDNFs), vascular endothelial growth factors (VEGFs), fibroblast growth factors 2 (FGF-2), and insulin growth factor 1 (IGF1) that are involved in depression. The BDNF growth factors are necessary for synaptic networks [31,32]. Depression is negatively correlated with expression of BDNFs in patients’ blood. The enhanced stress and depression, decrease the concentration of BDNFs in blood in the prefrontal cortex and hippocampus [33,34]. The reduction in neurotrophic growth factors are responsible to the disruptions in brain neuroplasticity caused by stress and depression [35]. Therefore, to overcome this situation, different antidepressants-like selective serotonin reuptake inhibitors (SSRIs) can be used to increase the levels of BDNF and modulate the neurotrophic growth factors expression. A frequent polymorphism of the BDNF gene (val(66)met) is suggested to modulate hippocampal neuronal plasticity and has been associated with individual variations in emotional reactivity traits and episodic memory [36]. Moreover, alleles (val(66)met) block the process of releasing of BDNF causing the shrinkage of neurons in the hippocampus and MPFC [37]. Furthermore, decreased levels of BDNF decrease the spine densities and dendritic lengths in hippocampus and PFC neurons and decrease volume of hippocampus [38,39,40,41].

Additionally, neurotropic factors are also linked with intracellular pathways and control the synaptic connectivity and neural function through the activation of Phosphoinositide 3-Kinase (PI3K), AKT, Raf-1 Proto-Oncogene, Serine/Threonine Kinase (RAF), Mitogen-activated protein kinase (MEK), and Extracellular signal-Regulated Kinases (ERK), respectively [42,43]. These signaling pathways relate to many downstream targets which affect many neuronal functions including protection and survival of neurons and initiation of synaptic plasticity. 

Rapamycin targets the protein kinase forming the mammalian Target Of Rapamycin (mTOR) that regulates the cell growth, survival, metabolism, and immunity. The mTOR exists in two forms mTORC1-2, which is formed by binding to two distinct accessory proteins such as Raptor and Rictor, respectively, with diverse substrates. The mTORC1 is an important kinase that activates the translation of synaptic proteins and is involved in regulation of synaptic plasticity [42,44]. The mTORC1 is composed of couple of downstream substrates like p70-S6 kinase 1 (S6K1) and eukaryotic initiation factor 4E binding protein 1 (4E-BP1). Moreover, activation of mTORC1 and enhanced synaptogenesis in the prefrontal cortex appear to be important in mediating the antidepressant effects of ketamine [45]. Therefore, faster-acting antidepressants stimulate the mTORC1signaling pathway in the prefrontal cortex [46,47,48,49]. 

Regulated in Development and DNA Damage Responses 1 protein (REDD1) is a conserved protein encoded by *REDD1* gene produced during cellular stresses [50]. The *REDD1* gene is present and mostly expressed in most adult tissues of human, mouse and drosophila, [51]. The REDD1 protein is composed of 232 amino acids which lack functional domains in the structure. The prior reported studies showed that REDD1 plays either a pro- or an anti-apoptotic role during the stress response, depending on the cell context [52]. REDD1 is a negative regulator of mTORC1; the expression of REDD1 is elevated in prefrontal cortex (PFC) causing depression in subjects during hypoxic stress [53]. The mice studies also showed that the chronic stress can decrease the level of mTORC1 signaling proteins causing the loss of neurotransmitters in the PFC [54,55]. 

## 6. Role of Neurotransmitters in Depression

**Figure 2:**
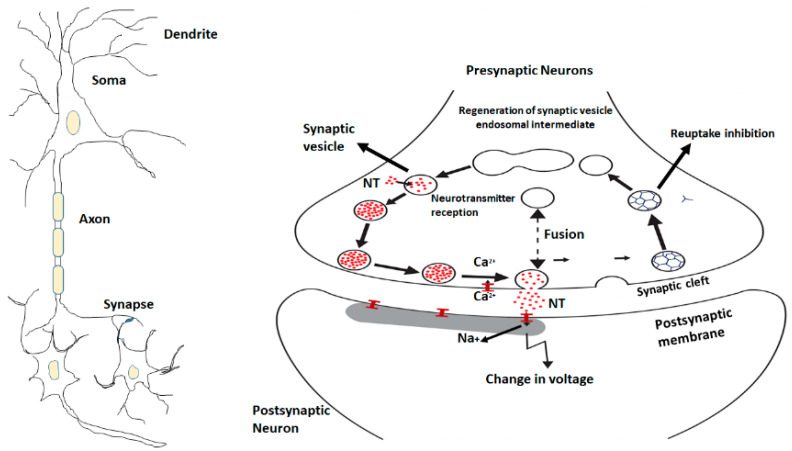
The neuronal signaling pathway and the movement of neurotransmitters through synaptic vesicles.

Neurosciences focus on brain regions (PFC, hippocampus, amygdala, anterior cingulate) and different neurotransmitters which control the regulation of normal emotions and stress-induced situations. Multiple neurotransmitters and neuromodulators like gamma-aminobutyric acid (GABA), glutamate, monoamines including serotonin, norepinephrine, dopamine, several neuropeptides are unique players in the regulation of depressive disorders and signaling pathways [56]. Most of the studies have reported biological abnormalities in parents with mood disorders but nowadays neurotransmitters, such as norepinephrine, dopamine, histamine, serotonin are the focus of research and theories of the etiology of these diseases. Disturbance of the signal neurotransmission system is the major reason to study the neurobehavioral system circuit and neuroregulatory mechanisms. Monoamines are key neuromodulators involved in brain functions. Norepinephrine and serotonin are the two most important neurotransmitters which are implicated in the pathophysiology of mood disorders [57] (Figure 2). 

### 6.1. Norepinephrine

Norepinephrine (noradrenaline) is monoamine neurotransmitter involved in depression [58]. Norepinephrine is functionally associated with multiple functions such as memory, stress reactions, and in emotions regulation. Norepinephrine acts as a hormone and is synthesized from dopamine by the catalytic reaction of β-hydroxylase. It has also been observed that norepinephrine producing neurons are mostly present in the pons and medulla parts of central nervous system (CNS). 

**Figure 3:**
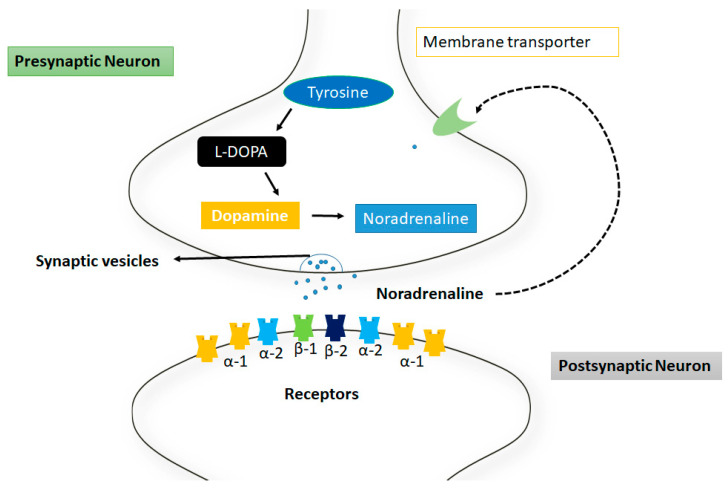
Mechanism of action of norepinephrine at synapsis. The tyrosine is converted into noradrenaline through intermediates such as L-dopa and dopamine respectively. These neurotransmitters are released from the membrane through synaptic vesicles. There are different receptors represented in different colors at postsynaptic neuron.

The norepinephrine neurotransmitters are usually used by the sympathetic nerves system and are found in the form of clusters in sympathetic neurons which are located near the spinal cord called sympathetic ganglia [59]. Norepinephrine acts on GPCRs, as adrenergic or adrenoceptors such as α-1,2 and β-adrenergic receptor, respectively and is frequently associated with arousal, alertness, and attention. However, the full extent of these actions is more complex and release in the sympathetic nerves system is typically associated with increased heart rate and blood pressure [60] (Figure 3). 

### 6.2. Serotonin

**Figure 4:**
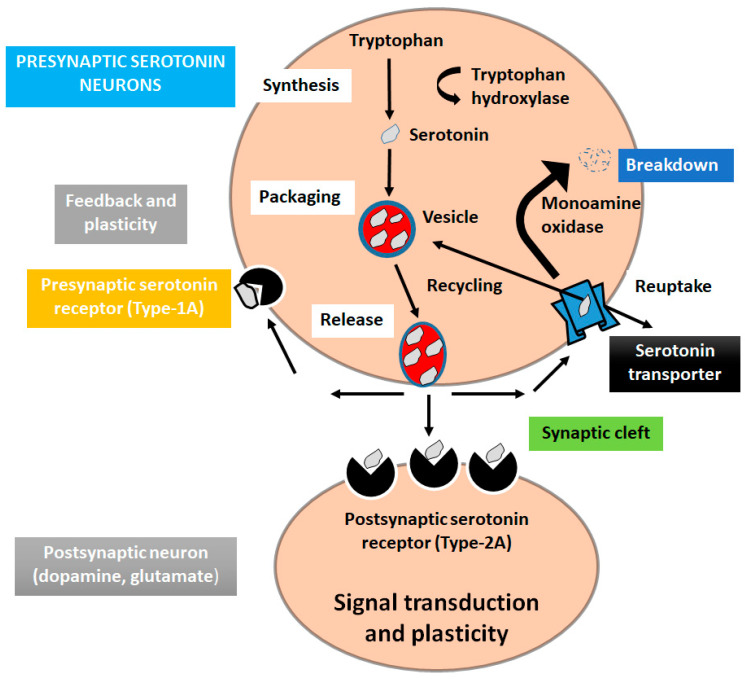
Serotonin signaling pathway in depression. The amino acid tryptophan is converted into 5HTP, and the 5HTP is converted into 5HT.

Serotonin (5-hydroxytryptamine) is a monoamine neurotransmitter involved in many brain functions modulating mood, and in different physiological processes [61]. Serotonin is produced in specialized neurons mostly in raphe nuclei and in major subdivision in the CNS [62,63]. To synthesize serotonin, the amino acid tryptophan is converted into 5HTP, and the 5HTP is converted into 5HT. There are seven different serotonin receptors (5-HT1, 5-HT2, 5-HT3, 5-HT4, 5-HT5, 5-HT6, and 5-HT7), which include six belonging to GPCRs and one associated with ligand-gated ion channels receptors [64,65]. Functional study showed that serotonin is associated with the pathophysiological depression and low concentration of serotonin in CSF may originate suicidal thoughts in patients. Therefore, many antidepressants are used to rise the levels of serotonin [66,67] (Figure 4). 

### 6.3. Dopamine 

Dopamine is a monoamine neurotransmitter involved in feeling pleasure, satisfaction and motivation [68]. Dopamine synthesis begins with the amino acid phenylalanine, and proceeds sequentially through tyrosine, DOPA, and then dopamine [69]. Dopamine is produced in the ventral tegmental area in the midbrain and acts as a messenger in brain. Dopamine works in various parts such as the PFC, amygdala, nucleus accumbens, hippocampus, and olfactory bulb in the brain. It has also been observed that dopamine pathways carry dopamine from areas of higher concentration to other parts of brain [70,71]. The bilateral dopaminergic pathway is a major dopamine nigrostriatal pathway in the brain that connects the substantia nigra pars compacta (SNc) in the midbrain with the dorsal striatum in the forebrain [72]. Another biochemical study showed that homovanillic acid (HVA) which is dopamine metabolite is responsible for dopamine turnover in depression patients. Moreover, the pharmacological treatments (antidepressants) have been reported for the treatment of depression [73]. 

### 6.4. GABA

γ-aminobutyric acid (GABA), a repressive neurotransmitter in CNS [74], that controls the anxiety and overactive fear circuits which are centered in the amygdala [75]. GABA is an amino acid that may help to improve mood [76]. Generally, GABA interacts with GABA-A and GABA-B receptors and mediates neuronal communication and functions and helps with feelings of anxiety, stress, and fear. It has been observed that the binding of GABA with GABA-A receptor is responsible for the permeability of negatively charged ions such as chlorides (Cl^−^) through the opening of ion channels [77,78]. The flow of chloride ions hyperpolarizes the membrane potential of the neurons and makes it less likely to fire an action potential. 

Whereas the GABA-B receptors causes the opening of potassium ion channels upon activation at the synaptic cleft. These channels allow positively charged potassium ions (K^+^) to flow out the neurons, again making the neuron hyperpolarized and less likely to fire an action potential [79]. These neuronal fluctuation and transmission of ions can control the depression and anxiety symptoms. Several studies showed that increased GABA levels in the brain can help to decrease anxiety, stress, and depression [80] and there are multiple drugs effects as alcohol and benzodiazepines which increase activity at GABA receptor [81,82]. 

## 7. miRNAs as Key Players in Depression

**Figure 5:**
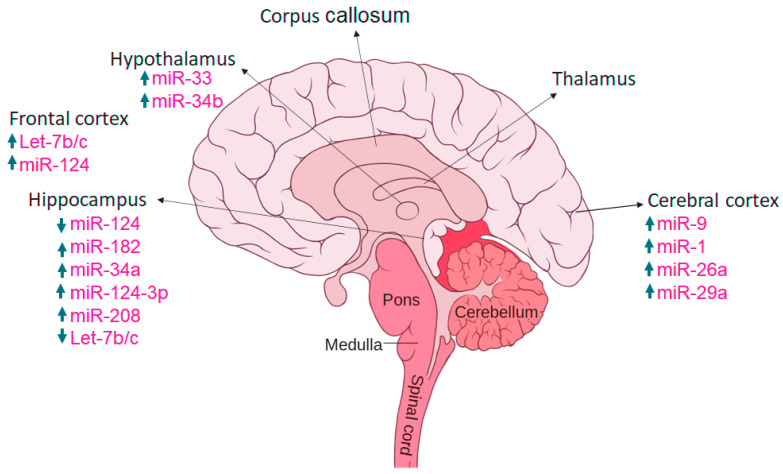
Expression of miRs in various brain regions, with arrows up and down indicating increased and decreased expression.

Many miRNAs are highly expressed in brain tissues and may be implicated in the pathological changes of the central nervous system (CNS) in depression (Shi et al., 2021). miRs are short (consisting of 19–24 nucleotides) and single-stranded RNAs which are genomically encoded to perform their function in gene expression and in post-transcriptional regulation of target genes [83]. These are non-coding single RNA molecules (that produce no proteins). miRNAs are found naturally, produced endogenously in cells. They are found in plants, animals, and in some viruses [84]. Gene expression performs its function after transcription when messenger RNA (mRNA) is formed. Expression controls if the production of a given protein is increased or decreased, generally the protein production is usually decreased. MicroRNA binds to the untranslated genes of messenger RNA, and there are two ways of binding; perfect complementary binding, and imperfect complementary binding. When there is a perfect binding the mRNA will degrade, and no protein is formed and when there is incomplete binding then the translation is repressed [85]. The overview of different miRs in various brain regions have been depicted in Figure 5.

### 7.1. miRNAs Control Neuronal Signaling Pathways

miRNAs are assumed to be a key players in the neuronal synaptic signaling [86,87]. The neurotransmitters such as glutamate, GABA, dopamine, and serotonin, are integrated into the presynaptic neurons, bundled into vesicles, and delivered into the post-synaptic neurons at synaptic cleft. This transportation of neuronal neurotransmitter is done by the encoded receptors present at the post synaptic neurons, encoded by mRNAs which are miRNA targets [88]. miR-153 regulates the synaptosomal-associated protein-25, (SNAP-25) [89] that mediates the calcium-initiated vesicle in combination with plasma layer and arrival of neurotransmitters in synaptic cleft [90]. Moreover, in model animal studies it was revealed that the overexpression of miR-153 represses the synaptic vesicle cycle at neuromuscular intersection whereas control expression results in antagonistic manner in the zebrafish. Furthermore, miR-153 also regulates the movement of zebrafish at undeveloped embryo stage which is probably the results of releasing the acetylcholine at neuromuscular intersections [90,91]. In another model animal study, it has been observed that miR-135a is directly involved in the regulation of early stress response. In the knockdown (KD) mouse study it was reported that miR-135a in amygdala induces an increase in anxiety-like behavior which was confirmed through spontaneous excitatory postsynaptic currents. Moreover, miR-135a also targets complexin-1 and complexin-2 (eukaryotic cytoplasmic neuronal proteins) which are significant for presynaptic vesicle combination [92]. 

The neuronal transportation across the presynaptic and postsynaptic neurons are impacted by different miRNAs through the calcium signaling at synaptic cleft. In another mice study, it was observed that miR-185 and miR-25 are depleted from mice to check their functional significance in brain. A couple of prior research showed that the deletion of miR-185 and miR-25 results in abnormally increase the level of sarco/endoplasmic reticulum Ca2+-ATPase (Serca2) which causes dysregulation of Ca^2+^ and glutamate in presynaptic terminals during the sustained neuronal activity that is required for synaptic plasticity at excitatory synapses. Therefore, serca2 levels are raised, which raises calcium levels in the presynaptic cytosol furthermore increasing synapse discharge [93,94]. 

A single miRNA might target numerous neurotransmitter receptors or subunits with restricting consequences for neuronal activity in the CNS [95]. miR-181a, is an smaller unit having great influence in the hippocampus-dependent memory formation [96]. Another report also showed that miR-181a targets both the α-amino-3-hydroxy-5-methyl-4-isoxazole propionic acid (AMPA) and the GABAAα-1 receptors, respectively and controls the mediating signaling pathways [97,98]. Overexpression of miR-181a in essential hippocampal neurons decreases the volume and thickness of dendritic spines, suggesting that miR181a may especially stop excitatory neurotransmission in neuronal cells [97]. Moreover, in vivo data also suggest that silencing of miR-181a produces neuroprotection against hippocampus neuron of cell apoptosis [99]. However, investigations also suggested that knowing a single target of a miRNA may not be adequate to predict the biological impacts of miRNA action on neuronal signaling [99]. 

The signaling impacts of numerous neurotransmitters are ended by reuptake of the neurotransmitter into the presynaptic transporters, and miRNAs might potentiate neurotransmission by restraining these transporters [100,101]. It has been observed that miR-16 targets SERT which ends serotonergic signaling and may acts as active player in the treatment of depression [102]. The specific serotonin reuptake inhibitor (SSRI) fluoxetine increases miR-16 and diminishes SERT expression in the raphe nuclei, and miR-16 overexpression copies the antidepressant impacts of fluoxetine in mouse models of depression. Then again, neurotransmission might be ended by enzymes that degrade the neurotransmission inside the synaptic split, and miRNAs that target these enzymes are anticipated to potentiate neurotransmission [102]. Similarly, miR-132 has a role in neuroplasticity function and may play an important role in depression [103]. miR-132 represses the formation of acetylcholinesterase (AChE) the catalyst (enzyme) that catalyzes the hydrolysis of acetylcholine at the neuromuscular intersection and in the CNS [104,105]. In mice study it has been observed that increased miR-132 levels in the hippocampus, decrease acetylcholinesterase levels, and impair execution of the hippocampus-dependent tasks. The disturbed expression level of miR-132 may cause neurological disturbance by regulating AChE [105]. Moreover, miRNAs can additionally target mRNAs that encode catalyst (enzyme) and cytoskeletal proteins that impact receptor surface expression, and degradation. For example, miR-125a regulates postsynaptic density protein 95 (PSD-95), a scaffolding protein that regulates the synaptic localization of many receptors, channels, and signaling proteins [106,107]. 

miR-146a-5p has significant role in neurological diseases and particularly acts as new potential therapeutic target for depression [108]. miR-146a-5p regulates the formation of microtubule-associated protein 1B (MAP1B) present in dendrites which regulates mGluR-induced AMPA receptor endocytosis and consequent long-term depression [109]. The overexpression of miR-146a-5p can block the mGluR-dependent AMPA receptor endocytosis and as a result it may be helpful in the depression. It has been observed that AMPA receptor endocytosis is increased without affecting GluA1 protein levels, and synaptic transmission is depressed by a MAP1B-dependent mechanism [108]. 

### 7.2. miRNAs and Growth Factor Signaling

Growth factors are group of proteins which control different metabolic processes through the activation and deactivation of downstream signaling pathways [110]. The clinical data showed that neurotrophic factor (BDNF), a neurotrophin growth factor involved in neuronal maturation, synapse formation and synaptic plasticity [111]. Moreover, the other vascular growth factors such as VEGF or VEGFA are involved in the possessing of neurotrophic and neuroprotective properties, respectively [112,113].

It has been observed that miRNAs straightforwardly target the mRNAs that encode growth factors [114]. The 3′UTR region of BDNF mRNA, which encodes BDNF, appears to be especially significant for regulating stimulus-induced BDNF formation in neurons [115]. A couple of miRNAs such as miR-26a and miR-26b target the 3′UTR of BDNF mRNA in HeLa cells and control the transcriptional activity of mRNA of BDNF [116]. Furthermore, miR-140 and miR-211 also play a similar function and bind with 3′UTR of BDNF in human astrocyte and regulate their inflammation-induced proliferation [117,118]. Another miRNA, miR-206 has good binding behavior with 3′UTR of mRNA of BDNF in mouse and represses BDNF protein formation in Neuro2A cells [119]. Another study shows that miR-149 inhibits the neuregulins (NRGs) signaling by targeting the mRNA that encodes the ErbB3 receptor [120,121]. These studies showed that miRNAs are key player in the regulation of growth factors which control the neurological behavior. Therefore, dysregulation of these miRNAs that target development factors may, add to neurological sickness and can provide a therapeutic target against brain related disorders. 

The tropomyosin receptor kinase B (TrkB) acts as receptor for BDNF and its binding alters the neuronal excitability of ion channels and enhances post-synaptic glutamate receptor activation causing influx of Ca^2+^ ions post-synoptically [122]. The mouse study showed that miR-592 levels decrease in the hippocampus following ischemic injury, permitting the quick synthesis of its target p75NTR, which thus advances apoptotic signaling and neuronal death [123]. 

### 7.3. miRNAs in Intrinsic Neuronal Excitability

The excitability of neurons is the ability to generate a large, rapid change of membrane voltage in response to a very small stimulus [124]. miRNAs can regulate inborn neuronal volatility by targeting ion channels that are voltage-gated, rather than ligand-gated. The miR-129 reduces the expression of the shaker voltage-gated potassium channel Kv1.1 encoded by the KCNA1 gene, and miR-324 restrains the synthesis of a Shal-type potassium channel Kv4.2. Both voltage-gated potassium channels Kv1.1 and Kv4.2 open in reaction to membrane depolarization and permit influx of potassium particles [125,126], and the loss of either channel leads to increased susceptibility to seizure in mouse models [127,128]. Both in vitro and in vivo studies showed that inhibition of miR-324-5p protects against kainic acid-induced downregulation of Kv4.2 in neurons. Restraining miR-324-5p fails to defer seizure in potassium voltage-gated channel subfamily D member 2 (KCND2) knockout mice. There is evidence that miR-324-5p plays a role in epileptogenesis via targeting of potassium channel Kv4.2. Some other miRNAs modify seizure susceptibility in mice and rodents by changing gene expression, which makes them attractive candidates as novel treatment targets in epilepsy [129]. It has been shown that reduced expression of miR-101 during early development in mice induced neuronal sensitivity later in life [130]. 

### 7.4. Role of miRNAs in Intracellular Signaling 

Ion channels proteins and receptors manage the neuronal signaling through membrane potential regulation [131]. miR-126 regulates the phosphoinositide-3-kinase (PI3K) downstream of insulin-like development factor 1 (IGF-1) by targeting the mRNAs that encode insulin receptor substrate 1 (IRS-1) and the PI3K administrative (regulatory) subunit p85β [132,133,134,135]. It has been observed that the hippocampal PI3K/AKT pathway mediates hydrogen sulfide (H_2_S) ameliorated depression and anxiety in diabetic rats by improving the hippocampal neurogenesis. Additionally, the inhibition of PI3K/AKT pathway by LY294002 exerted the improvement of hippocampal neurogenesis and the antidepressant and anxiolytic-like effects in the streptozotocin (STZ)-induced diabetic rats [136,137]. Another research study showed that, in neuroblastoma cell lines, the overexpression of miR-126 enhances IGF-1 functionality in cell expansion and increases sensitivity. The inhibition of miR-126 upgrades the defensive impacts of IGF-1 against neuronal cell death [135]. Furthermore, the overexpression of miR-126 in neurons diminishes the neuroprotective impacts of BDNF, nerve growth factor (NGF), IGF-1 and increases neuronal weakness to amyloid-beta (Aβ) to reduce neurotoxicity [138]. Besides, overexpression of miR-126 also decreases IGF-1 and induced Akt and p85β pathways [138]. These studies exhibited that miR-126 regulates various proteins downstream signaling pathways and directs neuronal responses to different growth factors. Another functional study showed that increased levels of miR-126 enhanced the functions of dopaminergic neurons signaling in cerebrum [139]. The miR-183 also focuses on mRNA which encodes mTOR and control translation [140]. The miRNA rise might contribute to motor neuron deficiencies in SMA, and stopes excess miRNA activities might have therapeutic utility. Additionally, miR-128 controls the downstream signaling of dopamine 1 receptors (Drd1) in motor neurons by targeting a few parts of the ERK1/2 pathway [141]. Moreover, loss of miR-128 in Drd1 positive neurons leads to motor hyperactivity and deadly seizures [118]. The ERK1/2 inhibitor rectifies ERK2 phosphorylation and motor hyperactivity in mice, showing a role of miR-128 in regulation of ERK1/2 signaling in controlling neuronal movement [142].

### 7.5. miRNAs and Neuronal Function

**Figure 6:**
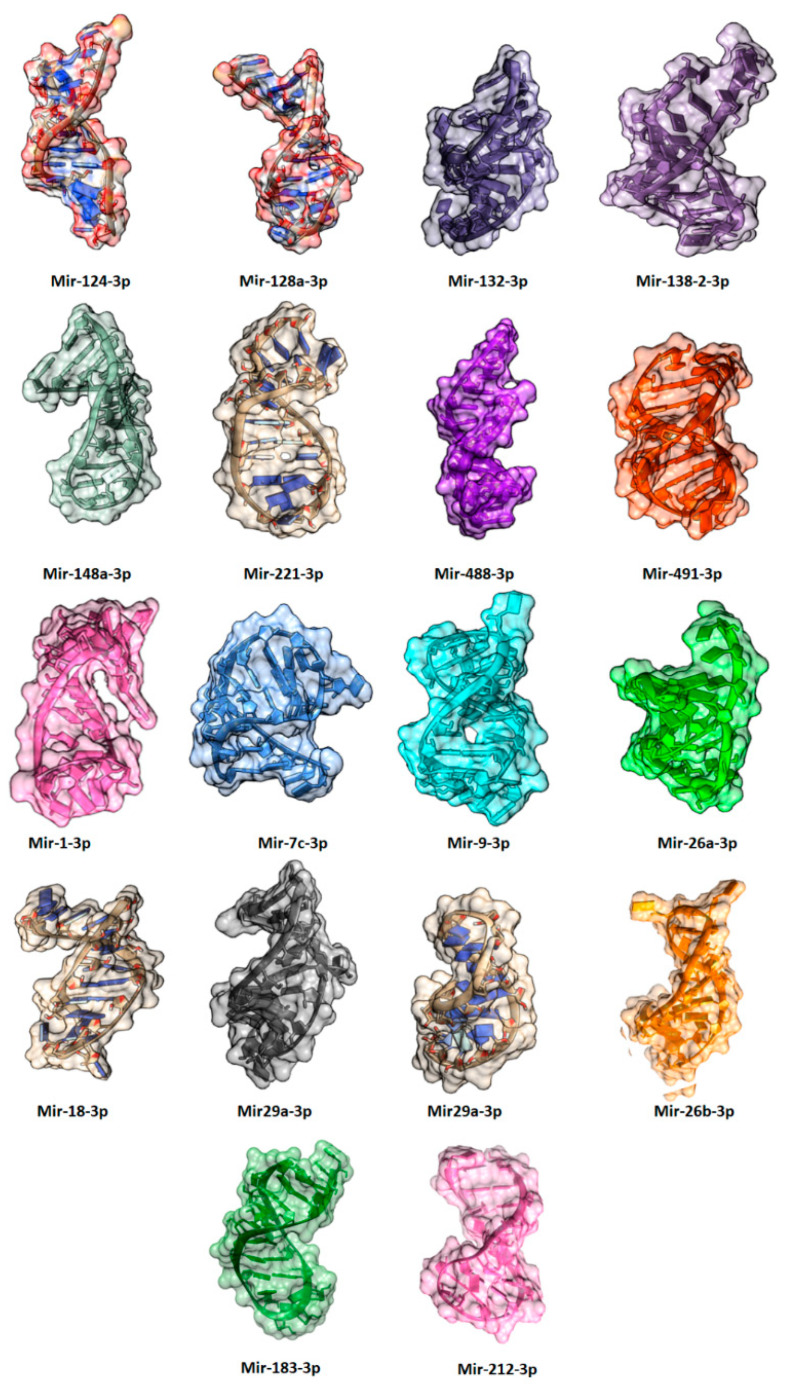
The predicted three-dimensional (3D) structures of miRNAs involved in anxiety and depression.

miRNAs play an essential role to regulate the genes which are involved in different stages of neurogenesis. There are many miRNAs involved in brain functioning some miRNAs are miR-132 which are involved in the formation of newborn neurons. miR-137 is used in proliferation and forming adult neural stem cells. miR-124 is the most abundant in mouse brain and involved in upregulation, differentiation and mature neurons. miR-9 is neuron-specific mainly expressed in the CNS that helps in cognition, learning, memory, and neuropsychiatric disorders [143]. miR-133b play important role in the maturation of dopaminergic neurons. Dopaminergic cells are the collection of neurons in CNS which can synthesize neurotransmitter dopamine which plays important role in multiple brain functions which include voluntary movement, behavior processes e.g., mood, addiction, stress. miR-18a is the negative regulator in neuronal cells, and it increases expression whereas, miR-16, miR-221, miR-204, miR-15b are highly expressed in distal axons. Recent evidence shows that members of the miR-34 family are the critical modulators for stress response involved in fear and anxiety-related behavior. The miR34c is upregulated in the central nucleus of the amygdala causing acute and chronic stress, therefore, could be used as antidepressant target [144] (Table 1) (Figure 6).

### 7.6. miRNAs and Stress Response 

Let us examine the role of miRNAs in response to different types of stress. Acute and chronic stress causes different changes in miRNA expression in brain areas [145]. Acute stress can cause an increase in expression of some selected miRNAs (such as miR-9, miR-26b, miR-29b, miR-30b, miR-30c, miR-30e, miR-125a, miR-126-3P, miR-129-3P, miR-207, miR-212, miR-351, miR-423, miR-487b, miR-494, miR-690, miR-691, miR-709, miR-711, and miR-7a-e) and induce a response in frontal cortex not in the hippocampus. Some of the miRNAs such as let7a, miR-9, miR-26a/b, miR-30b/c and miR-125a increase expression after acute stress. Physiological stress changes the expression of miRNAs in the central amygdala and hippocampus. Chronic stress causes more changes than acute stress, some miRNAs that were changed during acute and chronic stress are miR-132, let7a-1, miR-9-1, and miR124a-1. Prior studies showed that the overexpression of miR-124a is increased in the dentate gyrus and can exacerbate anxiety-like behavior target genes are BDNF [146]. 

miRNAs responses differently in stress-responsive brain regions. In the CA1 region, miR-376b and miR-208 shows increased expression, whereas, miR-9-1 depicts decreased expression in both acute and chronic stress conditions [147]. miRNAs are important in brain functioning they are involved in learning and memory processes and synaptic plasticity [148]. Moreover, some miRNAs are ubiquitously expressed e.g., let7b, miR-17-5P, and miR-21, respectively [149]. The expression patterns depend on the specific cell type and developmental stages [150]. miR-34a is brain and spinal cord specific, and miR-409-3p is involved in brain development in mice [151]. miR-9 and miR-124a are involved in neural lineage differentiation in embryonic stem cells derived culture [152].

### 7.7. miRNAs and Immune Response in Depression

miRs are also involved in the regulation of immunological responses including development, maturation, activation, functioning, and aging of various immune cells. Furthermore, miRNAs also exhibited highly specific expression patterns in organs associated with the immune system. miRNAs are also salient key players in both innate and adaptive immune responses [153]. Recent literature data suggested that miR-155, miR-146, and miR-223 exhibited significant role in the regulation of acute inflammatory response as well as in pathogen recognition. Multiple published data also reported that miRs are also involved in the adaptive immune responses which are characterized by activation and clonal expansion of T and B-cells. This activation and expansion lead to cytotoxic effector response and the production of antibodies in response to infections [154]. miRNA has been widely associated with modulating adaptive immunity by regulating the development, activation, survival, and proliferation of T- and B-cells [153]. Differential expression of miR-181, miR-17-92, miR-214, miR-146a, miR-155, let-7, miR-29, miR-125, and miR-216 has been observed in the signaling cascade downstream of T-cell activation [155]. Furthermore, higher expression of miR-150 is also observed in progenitor cells whereas mature B-cells represent its downregulation. Ectopic expression of miRNA-150 followed the premature downregulation of C-Myb, trigger apoptosis during the pro-B stage. Higher levels of miR-150 are necessary for the conversion of pre-B to mature B-cells (to downregulate C-Myb expression), guaranteeing normal B-cell development [156]. 

Moreover, there are multiple in vitro and bioinformatics studies which showed the significant role of miRs in depression and in suicidal attempts [157,158,159]. It has been observed that stress elicits a host of biological responses including neurochemical cascades in the hypothalamic-pituitary–adrenal (HPA) axis and immune reactions, which can subsequently alter neuronal connectivity and signaling as well as brain matter density [160,161,162,163,164,165]. There is no doubt about the involvement of miRs in the immune responses, however, it has been also shown that their abnormal expression in the immune system can be linked to multiple human diseases including inflammatory disorders, such as inflammatory bowel disease, and cancers [166]. miR-146a in turn inhibits expression of two components of the TLR4 signaling pathway, IL-1 receptor associated kinase and TNF receptor-associated factor-6 [167]. Thus, miR-146a functions as a negative feedback regulator of the TLR/NF-κB pathway. miR-155 and miR-146 expression is increased in macrophages in response to LPS stimulation, while miR-125b expression is decreased. miR-125b can target TNF-α mRNA, and a decrease in its expression leading to elevated TNF-α production and consequently increased inflammatory response [168].

### 7.8. miRNAs and Major Depressive Disorders

Major Depressive disorder (MDD) is psychiatric mood disorder and in severe conditions, this syndrome is accompanied by either delusion or hallucination [169,170]. Depression is a mood disorder in which a person feels sad every time, feels guilty, or has hypochondriasis [171]. Prior data showed that multiple miRNAs such as miR-1 42-5p/3p, miR-494, miR-376a*, miR-496, and miR-369-3p, miR-23b, miR-27b, miR-24-1*, miR-34b* and miR-34c, miR-17* and miR-20a are significantly downregulated in the MDD [172]. Moreover, miR-134 has stage-specific effects on cortical development [148,173,174]. miR-134′s has also significant involvement in synaptic development and plasticity regulation [175]. Exosomal miRNAs have been suggested to participate in the pathogenesis of neuropsychiatric diseases. The published data showed that exosomes from patients with major depression caused depressive-like behaviors in mice with involvement of miR-139-5p-regulated neurogenesis. Therefore, exosomal miRNAs are promising targets for the diagnosis and treatment of depression related diseases [176]. Another study showed that, miR are good and promising therapeutic biomarkers and good targets for treatment of depression [177].

**Table 1:** Role of micro RNAs in anxiety and depression.

miRNAs	Expression/Functions	References
miR-183	upregulate expression in the amygdala during acute stress	[147]
miR-212	pre miRNA upregulated expression in the medial prefrontal cortex maternal separation	[178]
miR-24a	decrease expression in the hippocampus due to treatment with lithium, sodium valproate	[179]
miR-488	SNP associated with panic disorder (genetic variation).	[180]
miR-491	SNP associated with panic disorder	[180]
miR-9	upregulate expression in frontal cortex during acute stress	[145]
miR-9-1, 3	pre miRNA upregulate expression in the medial prefrontal cortex	[178]
miR-18	stress and depression	[181]
miR-1	upregulate expression in the amygdala during chronic stress and downregulate expression in the hippocampus during acute stress	[147]
miR-26a/b	during acute stress upregulate expression in frontal cortex	[145]
miR-29a	during maternal separation upregulate expression in the medial prefrontal cortex	[178]
miR-208	under acute or chronic stress upregulation of expression in the central amygdala region of hippocampus	[147]
miR-221	decrease expression in the hippocampus	[179]
Let-7b/c	upregulate expression in frontal cortex during acute stress. Downregulate in the amygdala after acute and chronic stress; increase expression in the hippocampus	[145,147,179]
miR-124	Upregulate expression medial prefrontal cortex during maternal separation	[178]
miR-124-1	pre miRNA upregulate expression in the medial prefrontal cortex during maternal separation	[178]
miR-128a	decreases expression in hippocampal due to treatment of lithium and sodium	[179]
miR-128b	regulate the formation of dear extinction memory in the prefrontal cortex.	[182]
miR-132	upregulate expression in the medial prefrontal cortex during maternal separation	[178]
miR-134	form synaptic plasticity in hippocampus	[183]
miR-138-2	SNPs associated with panic disorder	[179]
miR-144	decrease expression in the hippocampus due to treatment of sodium, lithium valproate	[179]
miR-148a	SNPs associated with panic disorder	[180]

## 8. Pharmacological and Therapeutical Treatments for Depression and Related Disorders

### 8.1. Involvment of Selective Serotonin Reuptake Inhibitors in Depression

Selective serotonin reuptake inhibitors (SSRIs) such as fluoxetine, sertraline, citalopram, escitalopram, and fluvoxamine etc., are commonly used antidepressants agents [184]. These inhibitors are used in both moderate to severe depression and are relatively safe and typically cause fewer side effects [185]. These SSRIs inhibit the reuptake of serotonin by blocking serotonin transporter which result in increasing levels of serotonin available to bind to postsynaptic receptors [186,187]. Another rat study showed that depression can be controlled through the inhibition of SERT protein via miR-16 and as a result it has inhibited the reuptake of 5-HT in the hippocampus and serum [188]. Withdrawal of SSRIs can result in temporary deficiency of synaptic serotonin which in turn may lead to unpleasant symptoms such as headache, nausea, vomiting, and sleep disturbance [189]. 

It has also been observed that some miRs can regulate bacterial growth and gene transcription while also modulating the gut microbiota composition, suggesting the importance of miRs in gut and brain health. However, there are different treatment and prevention strategies for neuropsychiatric diseases, such as physical exercise, diet, and probiotics, that can modulate the gut microbiota composition and miRNAs expressions. The recent findings of the potential roles of microbiota and miR on the neuropathology of depression and anxiety, and its potential as treatment strategies [190].

### 8.2. Norepinephrine Reuptake Inhibitors

Norepinephrine reuptake inhibitors (NRIs) are drugs that act as a reuptake inhibitor for the noradrenaline and epinephrine by blocking the action of the norepinephrine transporter (NET) at the synapses and between nerve cells [191]. Norepinephrine is a naturally occurring neurotransmitter, or brain chemical, in the CNS that increases alertness and reaction time. Moreover, norepinephrine also plays a significant role in a person’s mood. It has been observed that low levels of norepinephrine can result in physical and mental symptoms, such as anxiety, depression, changes in blood pressure and heart rate, hypoglycemia, migraine and sleeping problems. NRI drugs are typically prescribed to patients who would benefit from increased levels of norepinephrine. Therefore, the list of NRIs which can be used in depression related symptoms includes atomoxetine (Strattera) for attention-deficit hyperactivity disorder (ADHD) and bupropion (Wellbutrin, Forfivo and Aplenzin) used for major depressive disorder (MDD). 

### 8.3. Tricyclic Antidepressants

Tricyclic antidepressants (TCAs) are named due to their core chemical structure with connected rings [192,193]. TCAs mechanism of action is not straightforward as like SNRIs. TCAs are primarily inhibiting the reuptake of both serotonin and norepinephrine by blocking both transporters [194]. Therefore, it has also been seen that TCAs: including amitriptyline, amoxapine, clomipramine, desipramine, doxepin, imipramine, maprotiline, nortriptyline, and protriptyline are involved in the blockade of other receptors and are thought to be responsible for their side effects more than their antidepressant activity [195]. TCAs are mainly used for depression due to their broad mechanism of action, they also proved to be beneficial in the treatment of other medical problems for example amitriptyline and nortriptyline have been used for migraine prevention as well as treatment of neuropathic pain, on the other hand TCAs such as doxepin have been used for insomnia [196,197]. TCAs block cardiac sodium channels and produce effects like antiarrhythmic agents such as quinidine this ultimately can lead to cardiac conduction abnormalities [198,199,200].

### 8.4. Monoamine Oxidase Inhibitors

Monoamine oxidase inhibitors (MAOIs) are antidepressants drugs commonly used in depression [201]. MOAs are the mitochondrial enzymes that degrade monoamines such as serotonin and norepinephrine [202]. Monoamine oxidase (MAO) possesses two types A and B which are distributed in the brain, gut, and liver tissues [203]. The MAO type A MAO-A preferentially metabolizes serotonin, norepinephrine and dopamine while MAO-B metabolizes dopamine, therefore the inhibition of MAO-A is thought to be responsible for antidepressant effects of MAOIs [204,205]. The primary mechanism of action of MAOIs is to inhibit the activity of MAO and preventing the breakdown of monoamine neurotransmitters and increase their availability. The MAOIs such as isocarboxazid, phenalgine, and tranylcypromine are irreversible inhibitors of both types A and B which in turn makes them effective for the treatment of depression. Another MAOI is selegiline which is a selective inhibitor for MAO-B and has been effective in reducing symptoms of Parkinson’s disease. MAOIs could be a good choice as the first or second-line antidepressants. But in practice, they are usually a very last choice, because MAOIs show not only a high incidence of drug-drug interaction but also drug-food interactions. 

### 8.5. Atypical and Typical Antidepressants

Antidepressants are medications used to treat major depressive disorders, some anxiety disorders, some chronic pain conditions, and to help manage some addictions. Common side-effects of antidepressants include dry mouth, weight gain, dizziness, headaches, sexual dysfunction, and emotional blunting [206,207]. An atypical antidepressant is any antidepressant medication that acts in a manner that is different from that of most other antidepressants [208]. The most recommended atypical antidepressants are bupropion, mirtazapine, trazodone, nefazodone, vilazodone, and vortioxetine with slightly different mechanisms of action [209]. Bupropion is a weak norepinephrine and dopamine reuptake inhibitor used for depression and it is effective in reducing the nicotine cravings [210]. Mirtazapine is an α-2 receptor antagonist that blocks presynaptic α-2 receptor and increases noradrenergic and serotonergic neurotransmission. Moreover, mirtazapine also exhibits postsynaptic serotonin receptor blocking and antihistaminic effects [211,212]. Vilazodone is another antidepressant drug that has unique mechanism of action and partially stimulates serotonin receptors [213,214]. 

Another couple of drugs such as imipramine and trazodone are used for the treatment of generalized anxiety disorder (GAD) in controlled trials [215,216]. In other studies, it has been shown that imipramine is effective as chlordiazepoxide and reduces depression however, trazodone is less sedating but effective against GAD [217]. Venlafaxine is the first approved antidepressant drug for the treatment of GAD [218,219]. 

### 8.6. Lithium

Lithium is mood-stabilizing drug that has been used in medicine for a long time, initially it was prescribed for depression however, currently it is also being used for bipolar disorder [220,221]. However, it has also been observed that unfortunately lithium has a fairly narrow therapeutic index which means that minor changes in dose or its blood levels can lead to toxicity [222,223]. 

### 8.7. Pharmacological Interventions Used in Anxiety Treatments

Medications that are used in anxiety and various other disorders have been shown to modify levels of miRNAs in the brain, but miRNAs are not the original target [146]. It has been shown that long-term direction of various mood stabilizers led to change in the expression of miRNAs in rat’s hippocampus when compared with saline treatment [179]. Several miRNAs including miR-29a, let-7c, let-7b, mir-128a, mir24a, mir30c, mir34a, and mir221 show changes in expression levels in patients with chronic oral treatment with valproate (VPA) or lithium. These microRNAs show decreased expression in the hippocampus, but increased expression of mir-144. For treatment of some forms of anxiety, VPA has shown promise for beneficial treatment such as social anxiety disorder [224]. Investigation of miR-34a downstream target genes in hippocampus shows that lithium direction of miR-34a and VPA increases the expression of miR-34a target genes. The direction of mirR-34a precursor reduces the expression of metabotropic glutamate receptor 7 (GRM7) [225]. Studies show that various numbers of miRNAs are linked with related downstream effects of therapeutics drugs which may be commonly used in the treatment of anxiety and various other disorders. Antidepressant-like SSRIs are used as first-line treatment in various anxiety disorders [226]. It has been shown SSRIs increase the expression of miR-16 in raphe nucleus and decrease the expression in locus coeruleus in mice [102]. In raphe nucleus and locus coeruleus, the expression patterns are inversely related with the expression level of its target genes, which are serotonin transporters (SERT). Studies reveal that therapeutic effects of fluoxetine could be related to different expression levels of mir-16 in monoaminergic neurons which target SERT in locus coeruleus and raphe nucleus [102]. In a mouse model with PTSD, 1-month treatment of fluoxetine can reduce or decrease anxiety-related behavior and is related to long-term effects on the expression of microRNAs [227]. After 74 days the expression level of mir-1971 decreased in the prefrontal cortex in comparison to the control group. Studies have revealed that some other SSRIs show possible relation between their miRNA regulation and corresponding behavioral effects. Maternal separation and chronic stress increase anxiety and depression which are associated with decreased levels of mir-326 expression in striatum and nucleus accumbent [228]. In long-lasting treatment, mir-326 levels increased in both areas of the brain compared with those of non-stressed or saline-treated rats. Mir-135a targets the serotonergic system and is attached to the serotonin transporter and 5HT-1a receptor gene [144]. Overexpression of mir-135a in serotonergic neurons is related to decreased anxiety and depression-like behaviors and knockdown can increase anxiety and depression [144]. Enriched microRNAs in the brain can target the GRM4 gene which can decrease expression when treated with antidepressants [229]. It has been shown that miRNA1202 plays an important role in antidepressant action by modifying glutamatergic neurotransmission. Major depressive disorder is related to decreased expression of miRNA1202 in the prefrontal cortex and the blood [230]. Therapeutic medication targeting other neurotransmitters and hormonal systems different than the serotonergic system have been analyzed for the contribution of miRNAs candidates in their antidepressant activities. Treatment with antidepressant agomelatine, which can target melatonin receptor and tianeptine glutamatergic, adenosine, and narcotic system, produced standardized stress-induced changes in miR levels in the dentate gyrus including the expression levels of miRNA-181b, miR-9, and miR-411 among others [231,232]. Treatment with the corticotropin-releasing hormone receptor 1 (CRHR1 also known as CRF1) antagonist NBI-27914 reduced the anxiety-like behavior of mice in elevated plus-maze and open field tests. miR-34b targets the CRHR1 genes and it was shown that administration of agomir for mir-34b in the paraventricular nucleus in the hypothalamus results in decreased anxiety behavior [233]. To target GABAergic neurotransmission, which can cause changes in microRNA expression, GABA receptor agonist gaboxadol was directed, which led to decreased anxiety-like behavior and increased expression of mir33 in the dorsal hippocampus [146]. It has been shown that NMDA-agonist D-cycloserine (DCS) which acts like an agonist at low doses but has antagonistic features with high doses, can be used to treat anxiety-like disorders [234]. 

### 8.8. Mechanism of Action of Rapid-Acting Antidepressant Ketamine in the Medial Prefrontal Cortex

**Figure 7:**
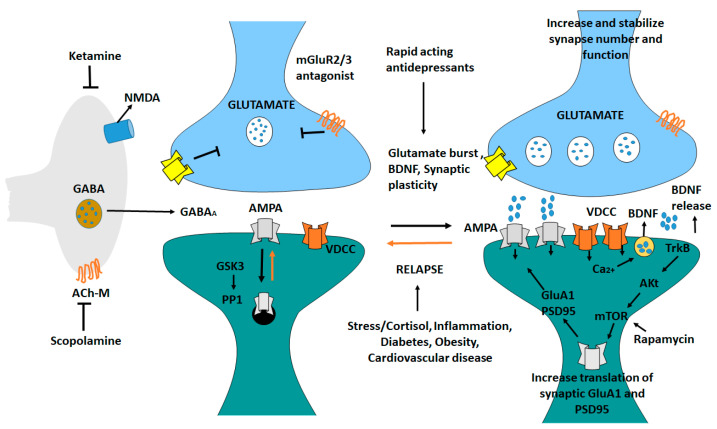
Antidepressant ketamine pathway. Ketamine causes a burst of glutamate which stimulates AMPA receptors causing depolarization and activation of voltage dependent Ca^2^+ channels, leading to release of BDNF and stimulation of TrkB-Akt that activates mTORC1 signaling leading to increased synthesis of proteins required for synapse maturation and formation. In other conditions where BDNF release is blocked the synaptic actions of ketamine are blocked. Scopolamine also causes a glutamate burst via blockade of acetylcholine muscarinic M1 (ACh-M1) receptors on GABA interneurons [239].

Ketamine generates a glutamate burst by disinhibiting GABAergic interneurons [235]. The tonic firing of these GABA interneurons is controlled by NMDA receptors, and the active, open channel state allows ketamine to enter and block channel function. The glutamate burst that follows activates AMPA receptors, resulting in depolarization and activation. BDNF is released and TrkB is stimulated because of the activation of voltage dependent Ca^2+^ channels [236]. This promotes mTORC1 signaling, increasing protein synthesis essential for survival, and development and creation of synapses (GluA1 and PSD95). BDNF is produced in situations where it is needed, the release is inhibited or neutralized by a mutant BDNF Val/Met knock-in mice or BDNF neutralizing antibody. When mTORC1 signaling is inhibited (e.g., by rapamycin infusion into the mPFC), synaptic and neuronal activity decreases. Ketamine’s behavioral effects are inhibited. Scopolamine also triggers a glutamate surge in GABA interneurons by blocking acetylcholine muscarinic M1 (Ach-M1) receptors [237]. Antagonists of glutamate metabotropic 2/3 receptors (mGluR2/3) also cause a rapid increase in glutamate levels (Figure 7). Blocking presynaptic autoreceptors, which inhibits the release of dopamine, has antidepressant effects [238]. Glutamate relapse into depression is linked to a loss of synapses in the mPFC [239]. Stress and endocrine (cortisol) and estrogen imbalances have been linked to the death of neurons. Inflammatory cytokines, metabolic, and cardiovascular diseases are all linked to inflammatory cytokines [240].

### 8.9. Pharmacotherapy and Psychotherapy for Generalized Anxiety Disorder 

The combination of pharmacotherapy and psychotherapy is used in the treatment of generalized anxiety disorder (GAD), which results in the reduction of symptoms, disability and improves health-related quality of life. Antidepressants, such as several benzodiazepines, buspirone, are used in the treatment of GAD. SSRIs and SNRIs are also used as first-line pharmacotherapies for generalized anxiety disorder [241]. Evidence is shown that SSRIs and SNRIs can be used for a treatment of anxiety disorder in children and adolescents [242]. Medication is provided to those children and adolescents only when the psychological approaches have failed. Control trials evaluated several psychotherapeutic techniques for GAD which include cognitive behavioral therapy, psychodynamic therapies, mindfulness-based therapies, relaxation therapy, with cognitive-behavioral therapy being the most successful [243]. Benzodiazepines are used for short-term relief of anxiety [244]. Evidence has shown that benzodiazepines are effective to reduce anxiety for a short period of time and there is no evidence that these drugs can work for a long period. As compared with other agents which were used to treat anxiety disorder, they are safe, rapid acting and have fewer side effects. Currently available benzodiazepines are effective for GAD. About 2/3 of patients experience improvement by using this drug within 1–2 weeks of treatment [215]. 

### 8.10. Obsessive-Compulsive Disorder and Diagnosis

The obsessive-compulsive disorder (OCD) is commonly chronic and long-lasting. In this disorder, a person has uncontrollable thoughts (obsession) [245,246]. Obsession means repetition of something in the mind which produces worry, and compulsive behavior in which the person repeats things, such as washing hands, cleaning home even if it is clean, etc. [247]. There are many types of neurotransmitters present in the brain, but serotonin is the most important neurotransmitter. When the levels of serotonin decrease in the body then the symptoms of obsessive-compulsive disorder increase. In our brain, there is a circuit which is the corticosteroid thalamic cortical circuit which is involved in connecting our lower body. Evidence is shown that if there is any dysfunction in this circuit then the symptoms of OCD are increased [248]. The combinatorial approach (medicines and psychotherapy) is important to cure these symptoms. Medicines which are selective serotonin reuptake inhibitors SSRIs are used to increase the level of serotonin. Some other medicines that can overcome anxiety for a short time are also used. The combination of medication and psychotherapy provides the best results [249]. 

### 8.11. Social Anxiety Disorder

This disorder, also known as social phobia it is a mental health condition [250]. It is an instant and continuous condition of fear when other people watch and judge the person. It is a chronic disorder. Onset occurs in children and adolescents (mid-teens) [251]. High-risk factors of social anxiety disorder are more prevalent in females as compared to males. Another factor is a family history of a social anxiety disorder (genetically or environmentally), shyness during childhood, behaviorally inhibited temperament, difficult unpleasant experiences during early childhood (child abuse or something that happened traumatically during childhood), and personality traits [252]. Any minor criticism is very seriously overreacted. These are associated frequently with other medical/psychiatric conditions such as major depressive disorder and substance abuse. Dysregulation of serotonin, or depletion of serotonin may increase the autonomic arousal by the increase of glutamate levels and increase of hypothalamic pituitary adrenal axis functioning which increases levels of stress hormones like activation of cortisol, and increased activity in the limbic fear circuit which increases activation of the amygdala [253]. The amygdala is the fear center in the brain. In adults fear or anxiety about one or more social situations include social interaction (conversation, meeting unfamiliar person), being observed (eating or drinking), performing in front of others (giving a speech). In children fear and anxiety occur in the situation of social interaction with other children but not with adults [254]. 

### 8.12. Social Anxiety Disorder and Medication

Usually, an individual with such disorder is fine with social interaction and shows only symptoms during performance situations. Treatment depends on social anxiety type, generally, we can use psychological treatments like cognitive behavioral therapy which could help in performance [255]. Pharmacologically we use selective serotonin reuptake inhibitors SSRIs (paroxetine, sertraline fluoxetine) serotonin-norepinephrine reuptake inhibitor SNRI (venlafaxine), or monoamine oxidase inhibitors MAOIs (phenalgine). In social anxiety disorder, performance type beta-blockers or benzodiazepines in an acute situation are used for short period (public speaking) [256].

### 8.13. Panic Disorder

Panic disorder is the type of anxiety disorder that causes panic attacks [257]. The person feels sudden terror even there is no real danger. The person feels losing control, with physical symptoms such as palpitations (an increase of heart rate), abdominal pain without any reason, nausea, intense fear of death, chest pain without any cardiac cause. Panic disorder is most common in students, it causes shortness of breath, trembling (shivering), sweating, and swearing [258]. For the patient with the acute condition, the benzodiazepine medication (BZD) is prescribed. This medicine is not for the chronic patient because BZD is highly addictive and has side effects. In chronic conditions, the selective serotonin reuptake inhibitor (SSRI) medication is prescribed together with psychotherapy (cognitive behavior therapy) which is highly effective for patient recovery. When the patient is facing a chronic condition increased amounts of panic attacks lead to depression, patient becomes agoraphobic being afraid of going into crowded areas, and having panic attacks in public. The frequent outcome of this disorder is substance abuse by taking alcohol, drugs and increased risk of suicide [259,260]. 

### 8.14. Bipolar Depression

Antidepressants can be divided into different classes such as selective serotonin reuptake inhibitors (SSRIs), serotonin/norepinephrine reuptake inhibitors (SNRIs), tricyclic antidepressants (TCAs), monoamine oxidase inhibitors (MAOIs) and atypical antidepressants [261]. Antidepressants differ in the way they work on presynaptic serotonergic neurons or serotonin-producing neurons, on the other hand presynaptic noradrenergic neurons or norepinephrine-producing neurons interact with corresponding postsynaptic neurons. The postsynaptic receptors of noradrenergic neurons are beta and alpha-1 while postsynaptic receptors of serotonergic neurons are serotonin 5-HT receptors; there are few subtypes of serotonin receptors present. Serotonin is synthesized from an amino acid tryptophan by serotonergic neurons and stored in vesicles [262,263]. On the other side norepinephrine is synthesized from an amino acid tyrosine by noradrenergic neurons and it is also stored in their vesicles for release. When serotonin and norepinephrine get released, they begin to stimulate the receptors and at the same time, they are transported from the synapse back to their neurons in a process called reuptake. Serotonin is reabsorbed by serotonin transporter (SERT) while norepinephrine is reabsorbed by norepinephrine transporter (NET) [264,265]. Once the serotonin and norepinephrine get reabsorbed back to their neurons they are partially repackaged into synaptic vesicles and partially broken down into inactive metabolites by an enzyme monoamine oxidase (MAO). 

## 9. Conclusions and Future Prospectus

miRNAs have been shown to play a variety of roles in the development, progression, and treatment of depression and the related disorders. In this review, we had highlighted multiple miRNAs such as miR15a, miR17-92, miR34, miR-101, miR-124, miR-135, and miR-155 (Table 1) that may act as key players in treatment of depression through activation and deactivation of signaling pathways. The collected data indicate that, peripheral miRNAs levels are prone to dysregulation such as miR-320a and miR-335 are significantly downregulated, while miR-451a and miR-124-3p are significantly upregulated in the depression patients. Controlling the expression of miRNAs is considered a possible way to treat depression, as this interaction is believed to be highly relevant. Therefore, interaction between of miRs and gene encoded proteins has led to the identification of signaling pathways like neurotrophic signaling, mTOR signaling, and PI3k/AKT involved in depression. In future studies, miRs could be used as new biomarkers for the treatment of depression by controlling different signaling pathways. 

## Data Availability

Not applicable.
